# Excessive Daytime Sleepiness in Obstructive Sleep Apnea Patients Treated With Continuous Positive Airway Pressure: Data From the European Sleep Apnea Database

**DOI:** 10.3389/fneur.2021.690008

**Published:** 2021-08-09

**Authors:** Maria R. Bonsignore, Jean L. Pepin, Fabio Cibella, Calogero D. Barbera, Oreste Marrone, Johan Verbraecken, Tarja Saaresranta, Ozen K. Basoglu, Georgia Trakada, Izolde Bouloukaki, Walter T. McNicholas, Sébastien Bailly, Athanasia Pataka, John A. Kvamme, Holger Hein, Stefan Mihaicuta, Ludger Grote, Francesco Fanfulla, ESADA Study Group

**Affiliations:** ^1^Sleep Disordered Breathing Clinic, Pulmonary Division, PROMISE Department, University of Palermo, Palermo, Italy; ^2^National Research Council (CNR), Institute for Biomedical Research and Innovation (IRIB), Palermo, Italy; ^3^HP2 Laboratory, U1042, INSERM, Grenoble Alpes University, Grenoble, France; ^4^Multidisciplinary Sleep Disorders Centre, Antwerp University Hospital and University Hospital Antwerp, Antwerp, Belgium; ^5^Division of Medicine, Department of Pulmonary Diseases, Turku University Hospital, Turku, Finland; ^6^Department of Pulmonary Diseases and Clinical Allergology, Sleep Research Centre, University of Turku, Turku, Finland; ^7^Department of Chest Diseases, Ege University Faculty of Medicine, Izmir, Turkey; ^8^Division of Pulmonology, Department of Clinical Therapeutics, School of Medicine, National and Kapodistrian University of Athens, Athens, Greece; ^9^Sleep Disorders Unit, Department of Respiratory Medicine, Medical School, University of Crete, Crete, Greece; ^10^Department of Respiratory and Sleep Medicine, School of Medicine, St. Vincent's Hospital Group, University College Dublin, Dublin, Ireland; ^11^Respiratory Failure Unit, G Papanikolaou Hospital, Aristotle University of Thessaloniki, Thessaloniki, Greece; ^12^Ear, Nose and Throat-Department, Foerde Central Hospital, Foerde, Norway; ^13^Private Practice and Sleep Lab for Internal Medicine, Pulmonary Medicine and Sleep Medicine, Geesthacht, Germany; ^14^Victor Babes University of Medicine and Pharmacy, CardioPrevent Foundation, Timisoara, Romania; ^15^Sleep Disorders Centre, Respiratory Medicine, Sahlgrenska University Hospital, Gothenburg, Sweden; ^16^Centre for Sleep and Wake Disorders, Institute for Medicine, Sahlgrenska Academy, Gothenburg University, Gothenburg, Sweden; ^17^Respiratory Function and Sleep Medicine Unit, Scientific Institutes of Pavia and Montescano, Istituti Clinici Scientifici Maugeri, Istituto di Ricovero e Cura a Carattere Scientifico (IRCCS), Pavia, Italy

**Keywords:** residual sleepiness, CPAP adherence, follow-up, Epworth Sleepiness Scale, sleep duration, sleep latency

## Abstract

Excessive daytime sleepiness (EDS) is a symptom of obstructive sleep apnea (OSA) that resolves under treatment with continuous positive airway pressure (CPAP). In some patients, sleepiness persists despite CPAP treatment. We retrospectively analyzed data on subjective residual EDS, assessed as an Epworth Sleepiness Scale score (ESS) >10, in patients from the European Sleep Apnea Database (*n* = 4,853, mean age ± SD 54.8 ± 11.8 years, 26.1% females), at baseline and at the first visit (median follow-up: 5 months, interquartile range 3–13). An ESS > 10 occurred in 56% of patients at baseline and in 28.2% of patients at follow-up. Residual EDS was analyzed in 2,190 patients (age: 55.1 ± 12.0 years, 26.1% females) with sleep monitoring data (median follow-up: 3 months, interquartile range 1–15). Sleep studies during CPAP use were obtained in 58% of these patients; EDS was reported by 47.2% of patients at baseline and by 30.3% at follow-up. Residual OSA, defined as an apnea–hypopnea index >10/h, and insufficient CPAP adherence, defined as nightly use <4 h, occurred with similar frequency in patients with and without EDS at follow-up. Prevalence of residual EDS was highest (40%) in patients with a first follow-up visit at 0–3 months, then it was 13–19% in patients with a first follow-up visit after 4 months to 2 years. The change in ESS (*n* = 2,190) was weakly correlated with CPAP use (R^2^ = 0.023, *p* < 0.0001). Logistic regression showed that an ESS score >10 at the first follow-up visit was associated directly with ESS at baseline and inversely with duration of follow-up, and CPAP use (R^2^ of the model: 0.417). EDS showed heterogeneity in different European countries both at baseline and at the first follow-up visit, suggesting modulation by cultural and lifestyle factors. In conclusion, residual EDS in CPAP-treated OSA occurred in approximately one in four patients at follow-up; its prevalence was highest (40%) in the first 3 months of treatment and subsequently decreased. The finding of residual EDS in a significant percentage of optimally treated OSA patients suggests that wake-promoting agents may be useful, but their indication should be evaluated after at least 3 months of treatment.

## Introduction

Obstructive sleep apnea (OSA) is characterized by complete or partial collapse of the upper airway during sleep, intermittent snoring, increasing inspiratory efforts, sleep fragmentation, and cyclic hypoxemia and hypercapnia (1). Excessive daytime sleepiness (EDS) is a major symptom of OSA, occurring in approximately a quarter to half of OSA patients (1, 2) and is associated with increased risk of accidents at work or while driving (3, 4). Continuous positive airway pressure (CPAP) is the most effective and commonly used treatment for OSA (5). CPAP application through a tightly fitted nasal or oronasal mask, at a sufficient pressure level titrated in each patient, prevents upper airway collapse and the pathophysiological consequences of OSA. CPAP treatment improves EDS either assessed subjectively by the Epworth Sleepiness Scale (ESS) or objectively by the Maintenance of Wakefulness Test (MWT) (5) and prevents sleepiness-related driving accidents (4).

The effect of CPAP treatment on EDS is multifactorial and conceptually linked to the improvement of sleep by alleviation of sleep disordered breathing. Despite a placebo effect (6, 7), all studies agree that therapeutic CPAP is more effective than subtherapeutic CPAP, not only on subjective but also on objective sleepiness measurements. The minimal important difference in ESS score associated with CPAP use has been estimated as a decrease in ESS score from baseline between two and three points (8, 9).

As expected, the CPAP-related improvement in EDS depends on adherence to treatment and differs according to the instrument used to assess sleepiness (10). In their seminal paper, Weaver and coworkers reported that CPAP treatment for 3 months resolved subjective EDS in 66% of adult patients classified as sleepy at baseline (ESS >10). The frequency of EDS resolution increased with CPAP use, but ~20% of the patients remained subjectively sleepy despite an 8-h nightly use of CPAP (10).

The persistence of EDS in OSA patients treated with CPAP may be affected by sleep duration, use of drugs, or comorbid conditions such as depression. After correcting for such confounding factors, a 6% prevalence of persistent EDS was estimated in CPAP-treated OSA patients after 1 year of treatment (11). Variable prevalence rates of residual EDS, between 13 and 40%, have been reported (12–14). Length of follow-up in these studies varied from 3 to 24 months (12–14). Each of these studies was conducted in a single country, and the potential influence of cultural or specific national conditions on residual EDS was not considered.

The purpose of this study was to assess the current prevalence of persistent EDS in CPAP-treated OSA patients in the large European Sleep Apnea Database (ESADA) cohort and to explore predictors among a large number of factors, including regional influences. Data were collected after a median CPAP treatment duration of 5 months.

## Patients and Methods

The ESADA has prospectively collected data in over 30,000 unselected adult patients (age 18–80 years) with suspected OSA syndrome studied in several European Centres of Sleep Medicine. A full description of the ESADA Database is available elsewhere (15). Briefly, collected data at baseline include anthropometrics, comorbidities, and use of drugs according to Anatomical Therapeutic Chemical Classification System codes. Sleep data, collected by polygraphy or polysomnography, include apnea–hypopnea index (AHI), oxygen desaturation index 3%, mean and lowest oxygen saturation (SpO_2_), and time spent at SpO_2_ <90% (T90); data on sleep stages were available only in patients undergoing polysomnography and were not considered in this analysis. Exclusion criteria in the ESADA cohort are previous diagnosis of OSA syndrome, limited life expectancy, and current alcohol or drug abuse. All patients provided written informed consent for the anonymous use of their data. Each study site obtained approval of the study by the local ethical committee.

**Figure 1:**
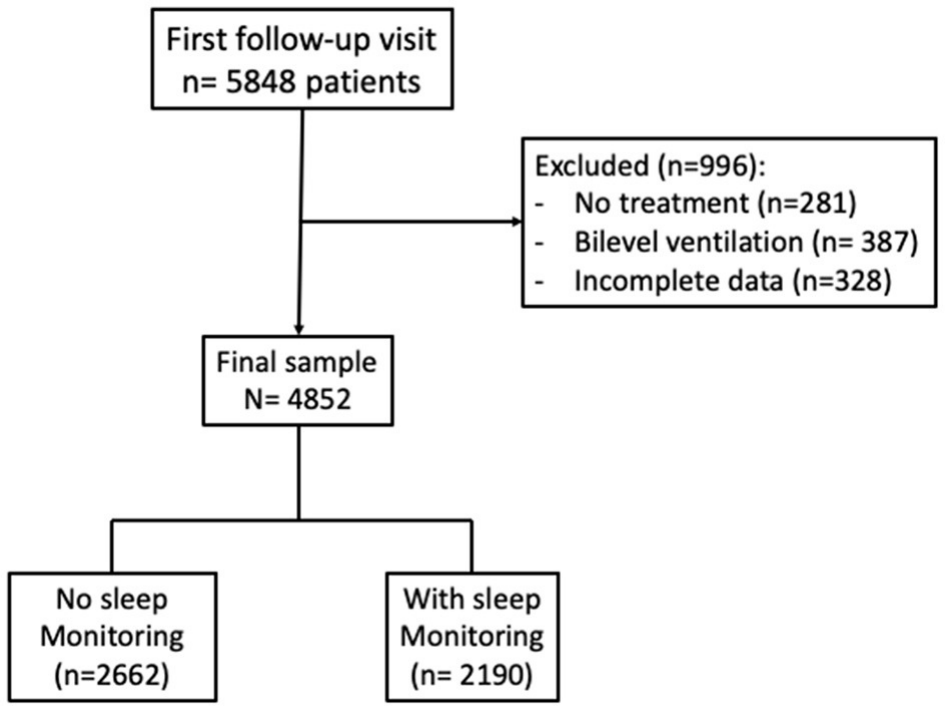
Flow-chart of the study.

Figure 1 reports the flowchart for inclusion in the study. Patients who received no treatment, or were treated with bi-level ventilation, or had incomplete data were excluded. Data obtained in patients on CPAP treatment at the first follow-up visit were analyzed to assess the prevalence of residual EDS and its predictive factors. Subjective EDS either at baseline or follow-up was defined as ESS score >10, an ESS score of 10 being the upper limit recorded in normal subjects (16). Sleep monitoring data at follow-up (residual AHI, mean hours of CPAP use) and their source (polysomnography, cardiorespiratory polygraphy, download of CPAP device) were recorded.

First, the prevalence of residual EDS was assessed on all patients with data at follow-up (*n* = 4,853). Then, a detailed analysis was performed in a subgroup (*n* = 2,190) with sleep monitoring data at follow-up (Figure 1). Sleep monitoring (PSG or PG) on CPAP was obtained in approximately 60% of this sample (Table 1). To account for possible causes of residual EDS on CPAP treatment, patients were stratified according to the occurrence of residual OSA, i.e., AHI ≤ 10 or >10/h, and the daily adherence to CPAP treatment, i.e., CPAP use <4 or ≥4 h. In addition, in each patient, the change in EDS status from baseline to follow-up was recorded, and four groups were defined and further analyzed: EDS at both baseline and follow-up; EDS at baseline and no EDS at follow-up; no EDS at baseline and EDS at follow-up, no EDS at either baseline or follow-up.

**Table 1:** Source of sleep monitoring data at the first follow-up visit in patients without and with residual excessive subjective sleepiness (EDS).

	**Total (*n* = 2,190)**	**EDS+ (*n* = 664)**	**EDS− *N* = 1,526**
Polysomnography, *n* (%)	103 (4.7)	36 (5.4%)	67 (4.4%)
Cardiorespiratory poligraphy, *n* (%)	1,405 (54.2%)	523 (78.8%)	882 (62.6%)
Limited channel study, *n* (%)	4 (0.2%)	2 (0.3%)	2 (0.1%)
Data download, *n* (%)	678 (31.0%)	103 (15.5%)	575 (40.8%)

*Statistical analysis:* Patients without and with EDS at follow-up were compared by unpaired t-test for continuous variables and by χ^2^ test for categorical variables. One-way analysis of variance or the Wilcoxon signed-rank test was used to compare baseline and follow-up data in the four groups defined according to the different combinations of EDS at baseline and follow-up. Significance was corrected for multiple comparisons. Logistic regression was used to analyze the factors associated with ESS > 10 at follow-up, based on results of this and previous studies (10–14, 17). The following variables were tested: age, sex, body mass index (BMI), ESS, AHI and comorbidities at baseline, subjective sleep latency at baseline, use of automatic or fixed CPAP, hours of CPAP use, subjective sleep duration at follow-up, and length of follow-up. Collinearity among variables was tested by Spearman rank correlation, and variables with Rho <0.70 were retained for analysis. Statview 5.0.1 (SAS Institute) and IBM SPSS Statistics Version 22 were used for analysis. Statistical significance was at *p* < 0.05 for all tests.

## Results

The study sample included 4,852 OSA patients on CPAP treatment at a first follow-up visit. The median follow-up duration was 5 months [interquartile range (IQR): 3–13 months]. Compared with baseline ESS score (median and IQR): 10 (6–14), median ESS score on CPAP treatment was four-point lower [post-CPAP: 6 (3–10), *p* < 0.0001]. Subjective EDS was reported by 56% of patients at baseline and by 28.2% at the first follow-up visit.

Sleep monitoring data during CPAP were available in 2,190 patients. In these patients, the median follow-up duration was 3 months (IQR 1–15). The prevalence of EDS at baseline (47.2%) was slightly lower than that found in the entire sample (56%). ESS score after CPAP treatment was three-point lower than at baseline [baseline: 10 (6–14), post-CPAP: 7 (4–12), *p* < 0.0001], and the prevalence of EDS at follow-up was 30.3%.

Compared with patients without sleep monitoring data (*n* = 2,662), patients with sleep monitoring data during CPAP treatment were of similar age and sex and showed slightly higher BMI; they reported longer subjective sleep duration and shorter sleep latency, besides showing a lower AHI, mean and lowest SpO_2_, and a similar percentage of time spent at SpO_2_ <90%. From a clinical point of view, however, the differences between groups were small (Supplementary Table 1). The group with sleep monitoring data showed a lower prevalence of type 2 diabetes, psychiatric disease, and drug treatment compared with the group without sleep monitoring data at follow-up (Supplementary Table 1).

### Analysis of Patients With Sleep Monitoring Data at Follow-Up (*n* = 2,190)

Polysomnography or cardiorespiratory polygraphy was obtained in 60% of the sample (Table 1). In patients with EDS at follow-up, data on residual AHI were collected from CPAP devices in 15.5% of cases. Most patients (81.1%) were treated with automatic CPAP (Supplementary Table 2). Compared with non-sleepy patients at follow-up, patients with EDS at follow-up were more often males, younger and more obese, showed slightly more severe OSA, and were sleepier at baseline, whereas comorbidities were similar in the two groups (Table 2).

**Table 2:** Differences at baseline between patients without and with excessive daytime sleepiness (EDS) at the first follow-up visit (*n* = 2,190).

**Variables**	**No EDS at follow-up (*n* = 1526)**	**EDS at follow-up (*n* = 664)**	***p***
Age (years)	55.7 ± 11.7	53.9 ± 12.4	0.0015^*^
Females, *n* (%)	27.6%	23.4%	**0.03**
BMI (kg/m^2^)	33.0 ± 6.6	34.2 ± 6.6	<0.0001^*^
AHI (events/h)	34.0 [21.0–51.4]	38.0 [23.0–60.0]	**<0.0001** ^‡^
ODI 3% (events/h)	31.0 [18.0–50.9]	38.1 [21.9–59.8]	**<0.0001** ^‡^
Lowest SpO_2_ (%)	77.1 ± 9.3	76.2 ± 10.0	0.029^*^
Mean SpO_2_ (%)	91.9 ± 3.3	91.4 ± 3.4	0.0032^*^
Time spent at SpO_2_ <90% (%)	3.9 [0.7–12.5]	5.3 [0.7–14.5]	0.159^‡^
ESS score	8 [5–11]	14 [12–17]	**<0.0001** ^‡^
Subjective sleep duration (h)	6.9 ± 1.4	6.7 ± 1.4	0.0017^*^
Subjective sleep latency (min)	10.0 [5.0–30.0]	5.0 [2.0–20.0]	**<0.0001** ^‡^
Coronary artery disease (%)	8.6%	7.1%	0.25^†^
Systemic hypertension (%)	49.6%	53.2%	0.12^†^
Type 2 diabetes (%)	12.4%	14.5%	0.19^†^
COPD (%)	7.0%	8.1%	0.33^†^
Asthma (%)	3.6%	2.3%	0.16^†^
Insomnia (%)	2.1%	1.4%	0.24^†^
Psychiatric disease (%)	4.8%	3.8%	0.28^†^
Follow-up duration (months)	5 [1–19]	1[0–7]	**<0.0001** ^‡^

* *one-way ANOVA (means±SD)*;

‡ *Mann-Whitney U-test test (medians and interquartile ranges)*;

† *χ^2^ test (percent)*.

*Significantly different variables are marked in bold*.

**Figure 2:**
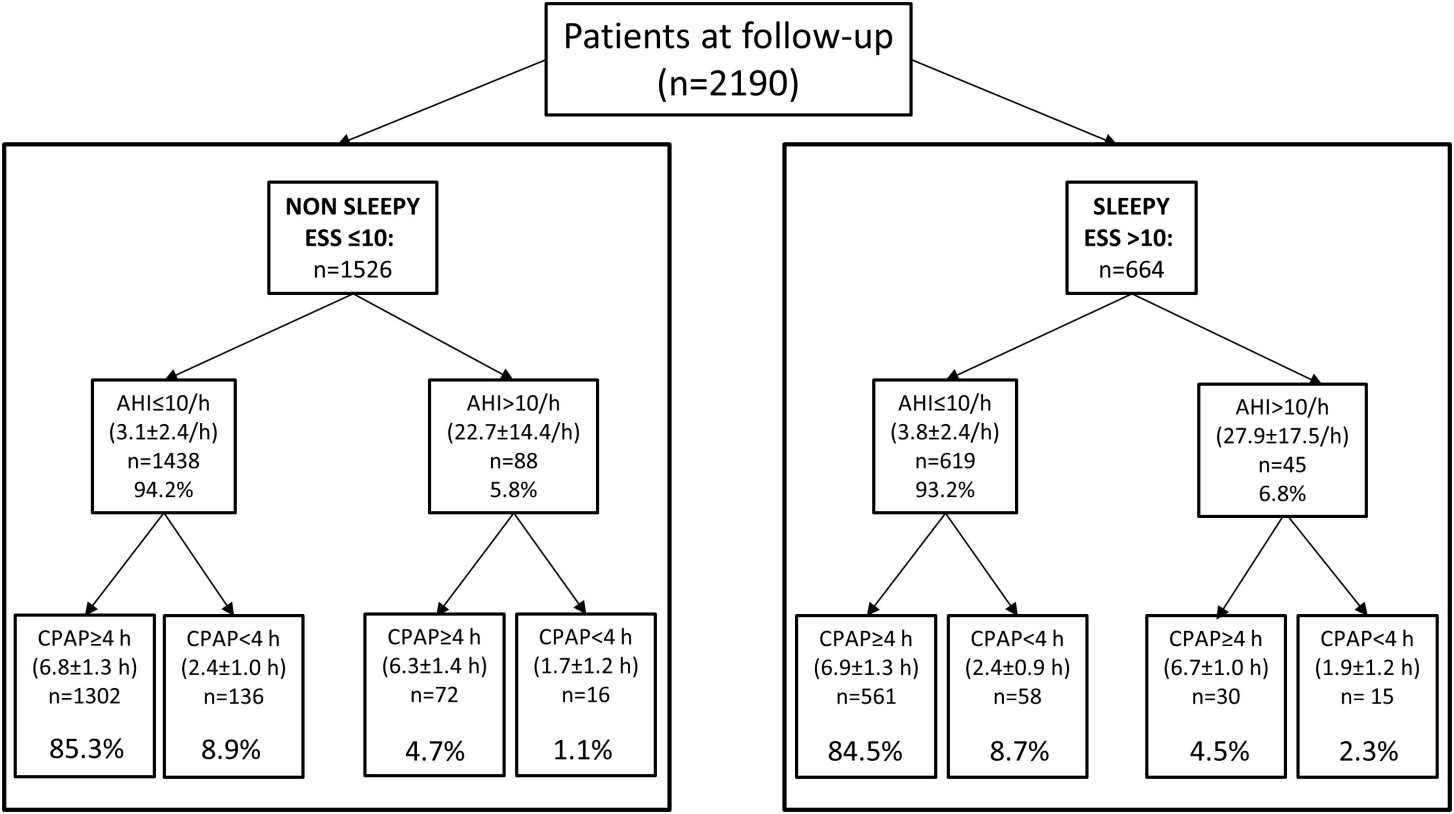
Analysis of the sample (sleep monitoring data available in 2,190 patients) according to resolution of OSA and adherence to CPAP treatment in patients without and with EDS at follow-up. Mean ± SD for AHI and CPAP use are reported in each box. Percentage of patients with incomplete OSA resolution or poor CPAP adherence was similar in patients with and without persistent sleepiness at follow-up (percentages in lower boxes refer to the non-sleepy and sleepy samples, respectively).

Patients were then stratified according to residual AHI (≤10 or >10/h) and adherence to CPAP treatment, i.e., CPAP use <4 or ≥4 h (detailed data are reported in Figure 2). The percentage of patients with incomplete resolution of OSA, i.e., AHI > 10/h, and poor CPAP adherence, i.e., <4 h/night, was 2.3% (15/664) in patients with EDS at follow-up compared with 1.1% (16/1,526) in patients without EDS at follow-up.

**Figure 3:**
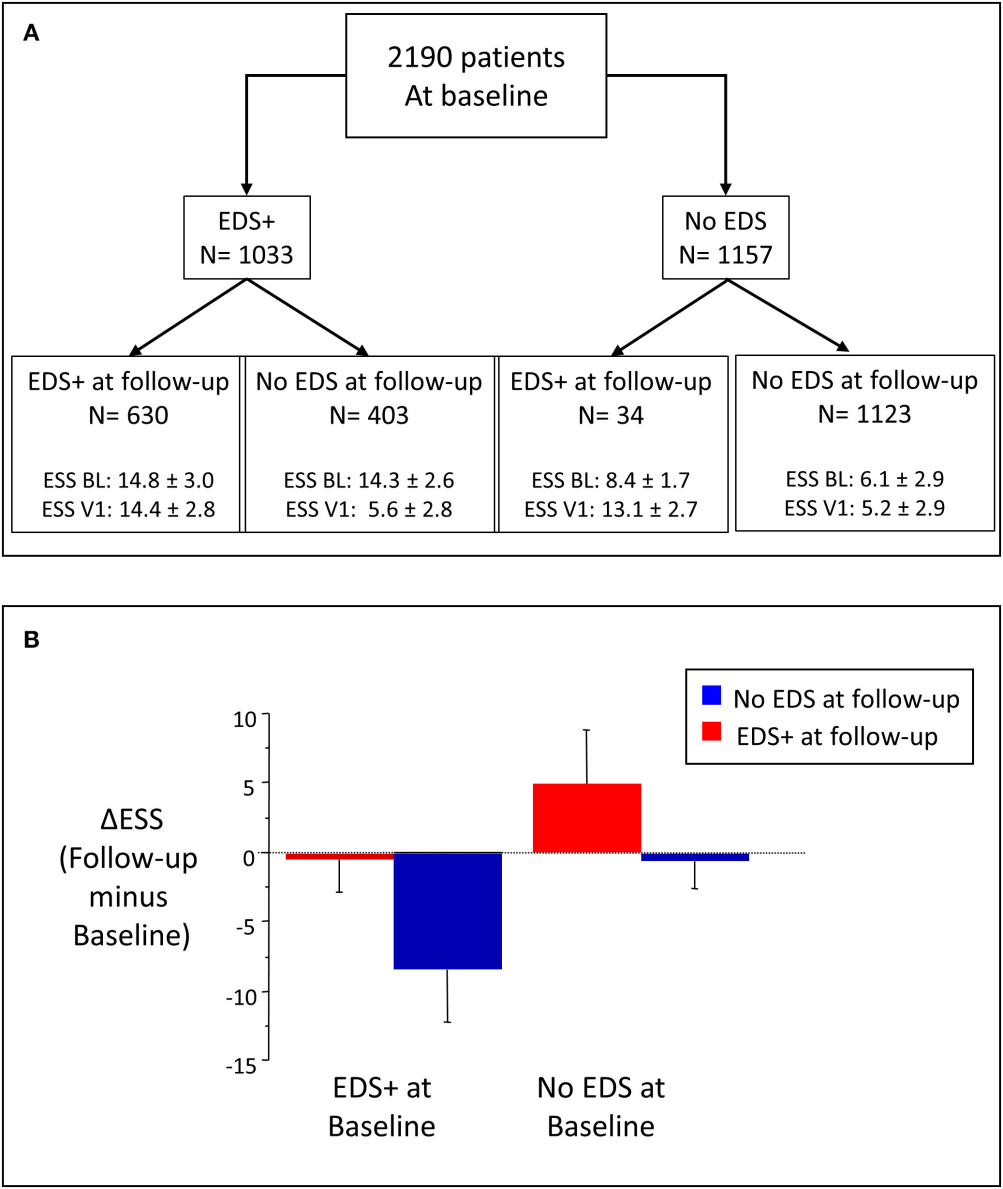
**(A)** Stratification of patients in four groups, according to occurrence of EDS at baseline and follow-up. EDS+: EDS present; BL: Baseline; V1: first follow-up visit. **(B)** Change in EDS (Δ EDS) in the four groups; Δ EDS calculated as EDS at follow-up minus EDS at baseline.

ESS scores were similar in sleepy patients at baseline, independent of the occurrence of EDS at follow-up (Figure 3A). A large decrease in ESS was found in sleepy patients at baseline in whom EDS resolved with CPAP treatment (Δ ESS = −8.7 ± 3.8, *n* = 403). Very few (*n* = 34) of the non-sleepy patients at baseline (*n* = 1,157) reported EDS at follow-up (2.9%, Δ ESS = 4.7 ± 3.9) (Figure 3B).

At baseline, patients with persistent EDS at follow-up were more obese than all the other groups and showed the highest prevalence of type 2 diabetes and the lowest prevalence of asthma; time to follow-up was also significantly shorter than in all the other groups (Table 3). Patients with EDS at follow-up showed the highest AHI and oxygen desaturation index 3%, and lowest SpO_2_ variables at diagnosis compared with the other groups; their median subjective sleep latency at baseline was 5 min compared with values ≥10 min in the other groups. Subjective sleep latency remained low in patients with persistent EDS at follow-up (Table 4).

**Table 3:** Clinical data at baseline according to occurrence of excessive daytime sleepiness at baseline and at the first follow-up visit, *n* = 2,190.

**Variables**	**EDS baseline/ EDS follow-up (*n* = 630)**	**EDS baseline/ No EDS follow-up (*n* = 403)**	**No EDS baseline/ EDS follow-up (*n* = 34)**	**No EDS baseline/ No EDS follow-up (*n* = 1,123)**	***p*-value**
Age (years)	53.6 ± 12.4	53.6 ± 11.8	59.2 ± 10.7^#^	56.4 ± 11.6	<0.0001^*^
Females (%)	23.3	25.1	20.6	28.1	0.13^†^
BMI (kg/m^2^)	34.4 ± 6.6	32.7 ± 6.4	30.8 ± 5.4	33.1 ± 6.7	<0.0001^*^
ESS score at baseline	14.0 [12.0–17.0]	14.0 [12.0–16.0]	9.0 [7.0–10.0]^§^	6.0 [4.0–9.0]^§^	**<0.0001^‡^**
Coronary artery disease (%)	6.5	9.3	17.6	8.3	0.07^†^
Systemic hypertension (%)	53.1	50.8	55.9	49.2	0.42^†^
Type 2 diabetes (%)	14.9	9.3	5.9	13.6	**0.03^†^**
COPD (%)	8.1	5.5	8.8	7.5	0.44^†^
Asthma (%)	2.2	7.0	5.5	2.7	**0.002^†^**
Insomnia (%)	1.3	2.3	2.9	2.1	0.58^†^
Psychiatric disease (%)	3.8	7.5	2.9	3.8	**0.015^†^**
Follow-up duration (months)	1 [0–5]	16 [7–30]^§^	14.5 [6.0–43.0]^§^	2 [0–12]^§^	**<0.0001^‡^**

* *one-way ANOVA, means and SDs*;

† *χ^2^ test (percent)*;

‡ *Kruskal-Wallis test (medians and interquartile ranges)*.

§ *statistically different compared to the group with EDS at baseline and at first follow-up visit*;

# *different from groups with EDS at baseline*.

*Significantly different variables are marked in bold*.

**Table 4:** Sleep monitoring data at baseline and at the first follow-up visit according to occurrence of excessive daytime sleepiness (EDS) at baseline and follow-up.

**Variables**		**EDS baseline/ EDS follow-up (*n* = 630)**	**EDS baseline/ No EDS follow-up (*n* = 403)**	**No EDS baseline/ EDS follow-up (*n* = 34)**	**No EDS baseline/ No EDS follow-up (*n* = 1,123)**	***p*-value**
Nocturnal monitoring:	BL					–
PSG (*n*)		52	71	8	125	
PG (*n*)		578	332	26	998	
limited channel (*n*)		0	0	0	0	
Nocturnal monitoring:	FU					–
PSG (*n*)		31	25	5	42	
PG (*n*)		512	100	11	782	
limited channel (*n*)		2	1	0	1	
CPAP download (*n*)		85	277	18	298	
AHI (events/h)	BL	38.3 [23.0–60.4]	35.0 [18.2–53.9]	24.9^§^ [13.3–37.4]	33.1^§^ [21.3–51.0]	**<0.001^‡^**
	FU	3.9 [2.3–5.8]	2.1^§^ [0.9–4.4]	1.9 [1.0–4.8]	3.1^§^ [1.4–5.1]	**<0.0001^‡^**
ODI 3% (events/h)	BL	39.2 [22.5–60.7]	30.2^§^ [15.6–51.0]	17.9^§^ [12.8–36.6]	31.0^§^ [19.4–50.8]	**<0.0001^‡^**
	FU	4.2 [2.8–6.3]	2.9^§^ [0.9–7.0]	3.6 [1.3–17.3]	3.6^§^ [2.1–5.5]	**<0.0001^‡^**
Lowest SpO_2_ (%)	BL	75.9 ± 10.0	76.1 ± 10.1	80.9 ± 8.4^§^	77.5 ± 8.9^§^	0.0002^*^
	FU	86.0 ± 6.8	82.9 ± 15.2^§^	86.9 ± 6.0	86.3 ± 8.8	0.003^*^
Mean SpO_2_ (%)	BL	91.4 ± 3.4	91.9 ± 3.5^§^	92.4 ± 3.6^§^	91.9 ± 3.2^§^	0.009^*^
	FU	93.8 ± 4.5	91.2 ± 16.1	95.5 ± 1.4	93.5 ± 8.4	0.02^*^
Time spent at SpO_2_ <90% (% of recorded time)	BL	5.4 [0.7–14.7]	5.3 [1.6–21.2]	3.0 [0.7–10.0]	3.6 [0.6–11.5]	**0.025^‡^**
	FU	0.2 [0.0–1.4]	0.1 [0.0–2.1]	0.0 [0.0–0.7]	0.1 [0.0–0.9]	0.081^‡^
Subjective sleep duration (h)	BL	6.7 ± 1.4	7.0 ± 1.4^§^	7.0 ± 1.2	6.9 ± 1.4^§^	0.002^*^
	FU	6.7 ± 1.4	7.0 ± 1.2	6.8 ± 1.2	6.7 ± 1.3	0.046^*^
Subjective sleep latency (min)	BL	5.0 [2.0–25.0]	10.0^§^ [5.0–30.0]	15.0 [5.0–16.3]	10.0^§^ [2.0–30.0]	**<0.0001^‡^**
	FU	5.0 [2.0–20.0]	10.0^§^ [7.0–20.0]	10.0 [2.0–15.0]	10.0^§^ [2.0–30.0]	**<0.0001^‡^**

*BL, Baseline, FU, first follow-up visit; PSG, Polysomnography; PG, Cardiorespiratory Polygraphy*;

* *one-way ANOVA, means and SDs*;

‡ *Kruskal-Wallis test (medians and interquartile ranges)*,

§ *different from the group with EDS at baseline and first follow-up visit*.

*Significantly different variables are marked in bold*.

**Figure 4:**
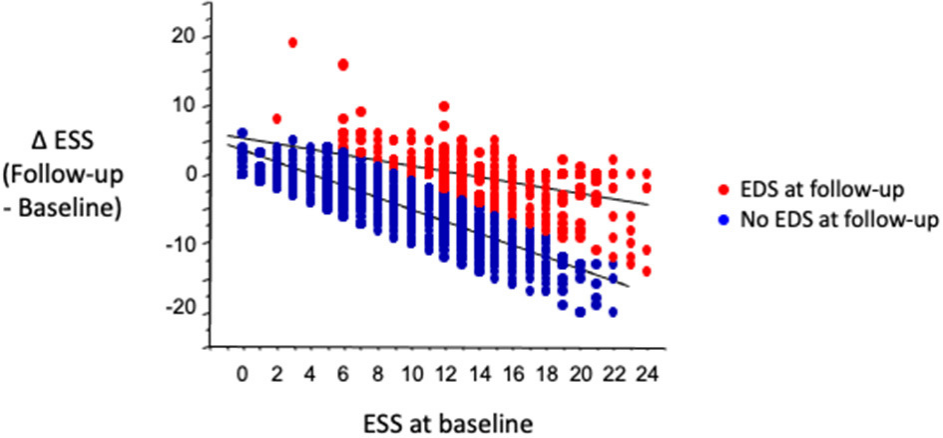
In patients sleepy at baseline, slope of relationship between change in ESS (Δ ESS, EDS at follow-up minus EDS at baseline) and baseline ESS was much lower in patients with than in patients without EDS at follow-up. Equations describing regressions are in patients with EDS at follow-up (red dots): ΔESS = 5.35 – 0.41 • ESS at baseline; R^2^ = 0.29, *p* < 0.001; in patients without EDS at follow-up (blue dots): ΔESS: 3.35–0.80 • ESS at baseline; R^2^ = 0.68, *p* < 0.001.

**Figure 5:**
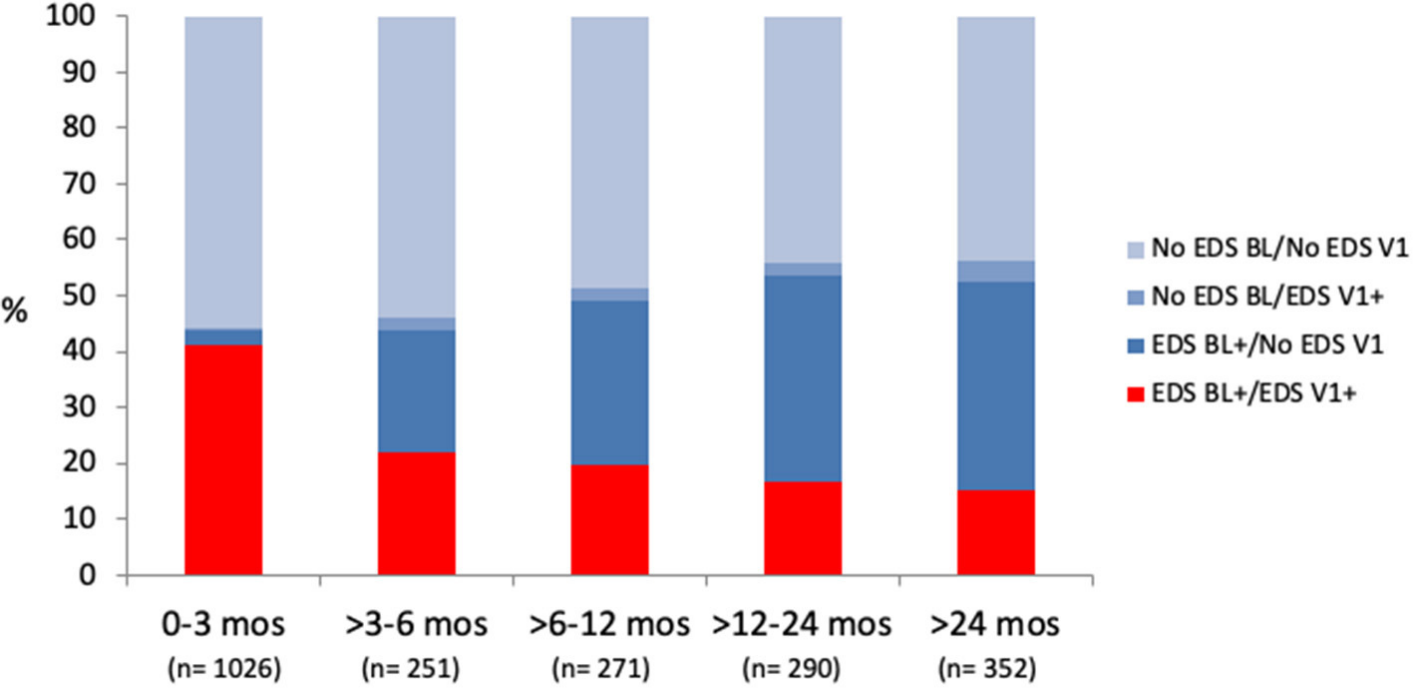
Distribution of four groups, stratified based on occurrence of EDS at baseline (BL) and follow-up (V1), according to time of first follow-up visit. Persistent EDS at follow-up (in red) was more frequent in patients seen during first 3 months of CPAP treatment (*p* < 0.0001 by X^2^, *p* for trend < 0.0001). No EDS BL/No EDS V1: ESS ≤ 10 at baseline and follow-up; No EDS BL/EDS V1+: ESS ≤ 10 at baseline and ESS > 10 at follow-up; EDS BL + /No EDS V1: ESS > 10 at baseline and ESS ≤ 10 at follow-up; EDS BL + /EDS V1+: ESS > 10 at both baseline and follow-up.

A weak relationship was found between hours of CPAP use and change in ESS (R^2^ = 0.023, *p* < 0.0001, *n* = 2,190). In patients with EDS at follow-up, the slope of the relationship between the change in ESS (Δ ESS, follow-up minus baseline) and baseline ESS was less than half the slope found in patients with resolution of EDS after CPAP treatment (Figure 4). The prevalence of EDS at follow-up was highest in sleepy patients at baseline undergoing the follow-up visit in the first 3 months of treatment; subsequently, the prevalence of persistent EDS decreased slightly over time, from 21.9% at 3–6 months to 15.1% at more than 2 years (Figure 5, *p* for trend < 0.0001).

Logistic regression was used to analyze the predictors of ESS score >10 at follow-up (*n* = 2190, R^2^ of the model: 0.417). The following variables were identified as correlates:

- ESS at baseline (0.139 [95% CI 0.015/0.263] *p* = 0.028)
- Hours of nightly CPAP use (−0.514 [95% CI −0.767/–0.261], *p* < 0.0001)
- Follow-up duration in months (−0.051 [95% CI −0.064/−0.039] *p* < 0.0001)
- Interaction ESS at baseline^*^hours of CPAP use/day (0.051 [0.031/0.071, *p* < 0.0001)

There was a trend for AHI at baseline (−0.006 [95% CI −0.013/0.001] *p* = 0.088), and average subjective sleep length at follow-up (−0.10 [95% CI −0.215/0.013], *p* = 0.084) to be inversely related with persistent EDS. Age, sex, BMI, subjective sleep latency, comorbidities, or use of fixed or automatic CPAP did not enter the model. In summary, EDS at follow-up was associated with high ESS at baseline, short nightly CPAP use, and short follow-up duration at first control visit. The highly significant interaction term indicates that a high degree of sleepiness at baseline reduced the effect of CPAP adherence on the outcome.

**Figure 6:**
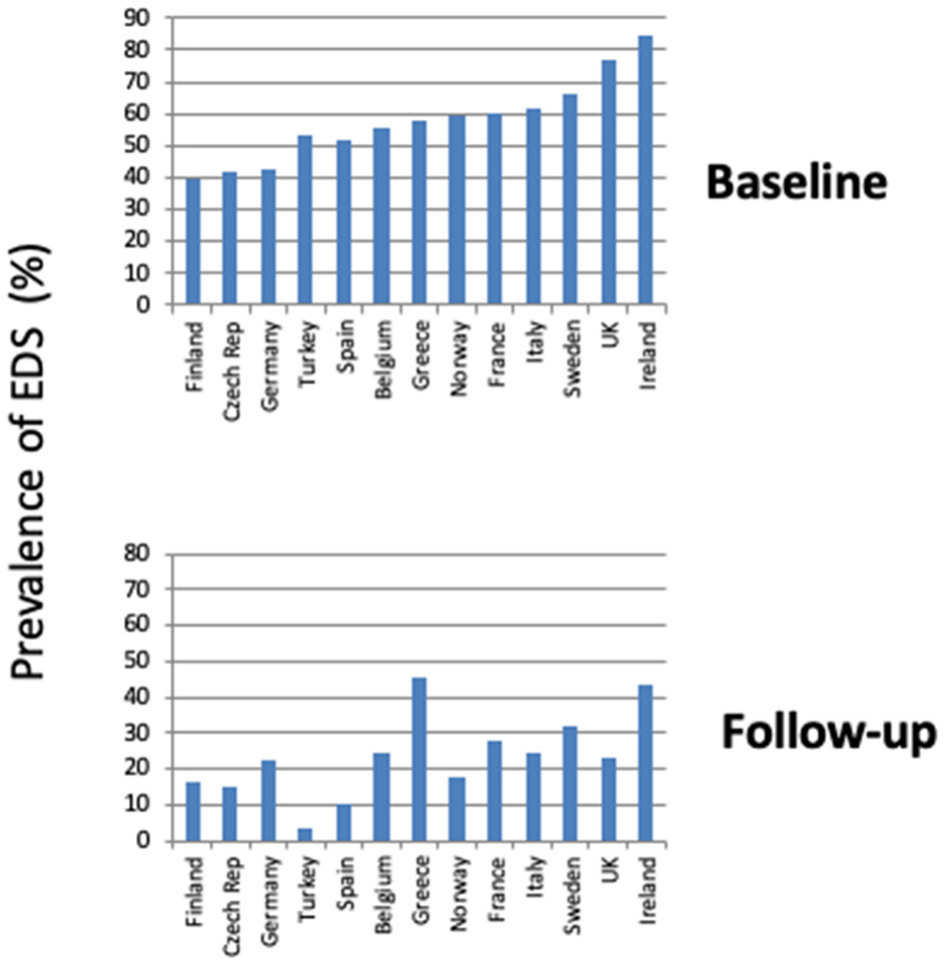
Prevalence of EDS at baseline and first follow-up visit in different ESADA countries in entire sample. Only countries reporting data on at least 50 patients are shown.

Finally, the geographic distribution of EDS was explored at baseline and follow-up in the entire sample (*n* = 4,853). This analysis was limited to countries with data on at least 50 patients (50–1,839) (Figure 6). Prevalence of EDS was highly variable, occurring in 39.8–84.8% of patients at baseline and in 3.6–45.6% of patients at follow-up. The highest prevalence rates of EDS were recorded in the samples from Sweden, United Kingdom, and Ireland at baseline and in the samples from Greece and Ireland at follow-up.

## Discussion

Approximately one in four patients treated with CPAP reported EDS at follow-up. The prevalence of residual EDS was highest when the follow-up visit occurred during the first 3 months of CPAP treatment. Incomplete resolution of OSA and/or poor adherence to CPAP were similar in patients with and without EDS at follow-up. The major predictive factors of residual EDS were baseline EDS, hours of CPAP use, and length of follow-up. At least part of the variability in EDS at follow-up might be related to geographical/cultural differences in patients' samples or subjective EDS reporting.

The analysis of CPAP-treated OSA patients from the ESADA cohort revealed a quite high prevalence of EDS at the first follow-up visit. The sample of treated OSA patients was large and representative of patients from several European countries. Importantly, sleep data at follow-up were collected during sleep studies on CPAP in 45% of the entire sample, and data downloaded from CPAP devices accounted only for 15.5% of patients with residual EDS in the sample with sleep monitoring data during CPAP treatment. Prevalence of EDS at follow-up was 28.2% in the entire sample, highlighting that EDS during OSA treatment is a clinically relevant, and often overlooked, problem. Residual EDS was more prevalent in patients assessed early during follow-up, then its frequency slowly decreased over time.

Compared with patients without EDS at follow-up, patients with persistent EDS were younger and more obese and showed slightly more severe OSA at baseline; they were sleepier at baseline and reported shorter sleep duration and sleep latency, in agreement with data reported in previous studies on EDS after treatment. In the study by Pepin and coworkers, patients with residual EDS were younger and sleepier at diagnosis, but the prevalence of EDS after 1 year of treatment was lower than in our study, being 12% in the overall sample, and only 6% after excluding patients with known causes of EDS, including restless leg syndrome and depression (11). In the study by Gasa and coworkers, persistent EDS during CPAP treatment was associated with baseline ESS score and was more prevalent in patients with moderate OSA; they also noted that a small number of non-sleepy patients at baseline became sleepy on CPAP treatment (5.6 vs. 2.9% in the current study) (12). As for the possible role of residual AHI or level of adherence to CPAP treatment, the prevalence of incomplete OSA resolution or poor CPAP adherence was similar in subjects with and without persistent EDS. Weaver et al. also reported no difference in the average use of CPAP between sleepy and non-sleepy OSA patients at 3-month follow-up (10). The prevalence of EDS decreased with increasing hours of CPAP use; however, ~20% of patients using CPAP for >8 h/night reported residual EDS (10). In the study by Antic and coworkers, 40% of patients with moderate–severe OSA reported EDS after 3 months of CPAP treatment (14). Our analysis took into account the length of follow-up, and, similar to previous results (10, 14), we found a high prevalence of EDS when the follow-up visit was in the first 3 months of CPAP treatment. We cannot exclude a selection bias, i.e., patients with persistent EDS may have asked for an early visit because of problems associated with CPAP treatment. We also acknowledge that we did not longitudinally assess patients at different time points during follow-up, and the higher prevalence of EDS in the first 3 months needs confirmation in future studies.

Because the sample included patients with and without EDS at baseline, we analyzed the longitudinal changes in ESS according to initial and final EDS status. Of 2,190 patients, 47.2% were sleepy at baseline (*n* = 1,033), and 61% of them (*n* = 630) were sleepy at follow-up. These figures may reflect a selection bias, i.e., sleep monitoring data would more likely be obtained in symptomatic than asymptomatic patients at follow-up. Patients with persistent EDS showed a very short sleep latency compared with the other groups both at baseline and follow-up. Although sleep latency is expected to be short in sleepy patients, our data suggest that a very short sleep latency could help in the clinical identification of patients more likely to remain sleepy on CPAP. However, short sleep latency did not enter the logistic model.

Incomplete resolution of EDS could reflect the individual susceptibility to hypoxic brain damage or sleep fragmentation. Convincing data on the pathogenesis of intermittent hypoxia-induced sleepiness and on the role of sleep fragmentation have been obtained in rodents. A loss of 40% of wake-promoting catecholaminergic neurons has been shown in rodents after chronic intermittent hypoxia exposure for 6 months (18). White matter damage (19) and possible vicious cycles of oxidative damage involving both neurons and microglia (20) have been reported after chronic intermittent hypoxia in mice. Similarly, neuronal damage was reported in wake active regions after 4 weeks of sleep fragmentation (21). In humans, the mechanisms preventing full recovery from EDS remain unknown, but imaging studies hold some promise, as abnormal findings in the frontal area (22) and white matter alterations (23) were found in patients with persistent EDS during CPAP treatment. In our study, ESS scores on CPAP decreased less in patients with persistent EDS than in patients without EDS at follow-up, for similar pretreatment ESS levels. This result agrees with the report by Gasa et al. (12) who found that some improvement in symptoms occurred with CPAP treatment in patients with persistent EDS at follow-up, albeit to a lesser extent than patients with no residual EDS.

A small group of patients not sleepy at baseline became sleepy on CPAP. These patients were older compared with the other groups. The low number of patients prevents any meaningful analysis, but this subgroup may show different clinical features compared with patients with persistent EDS, i.e., high prevalence of coronary artery disease or sleepiness due to adverse effects of CPAP.

The variable prevalence of EDS in different European countries both at baseline and follow-up may reflect the variability in several factors, including the local prevalence of obesity, cultural factors, and lifestyle habits, such as caffeine and alcohol consumption. This topic deserves further study.

As for factors associated with persistent sleepiness, EDS at follow-up tended to be negatively related to baseline AHI, in agreement with the results reported by Gasa et al. (12) who found that persistent EDS was associated with mild–moderate OSA rather than severe OSA. Obesity is a potential cause of daytime sleepiness, even in the absence of OSA (24). The role of obesity in the pathogenesis of EDS is supported by its improvement/resolution after weight loss, independently of changes in AHI (25). However, BMI did not enter the logistic model in our study.

Other studies have searched for predictors of persistent EDS in CPAP-treated OSA patients (12, 26). Our data add the important information that prevalence of residual EDS in CPAP-treated patients, after an initial high value during the first 3 months of treatment, similar to previous studies (10, 14), slowly decreased during the first 2 years of treatment. This finding opens the way to pharmacological treatment of EDS. New drugs, i.e., solriamfetol and pitolisant, have been recently studied in OSA patients and appear effective without major adverse effects (27–29). The indications to drug treatment are still undefined, but according to our results, it seems advisable that their prescription should occur at least after 3 months of CPAP treatment.

Our study shows some important limitations. First, sleep monitoring data were available in only 45.1% of patients undergoing the first follow-up visit. The analyzed subgroup of 2,190 patients showed a higher prevalence of EDS at follow-up than the entire sample, suggesting a selection bias. On the other hand, such an enrichment with a high percentage of persistently sleepy OSA patients on CPAP may increase the robustness of the analysis of predictors for persistent EDS. Moreover, the patients with and without sleep data at follow-up were similar for anthropometrics and OSA severity at baseline. Second, the analysis did not take into account the type of sleep study at baseline or follow-up. The ESADA cohort includes patients studied at baseline with either polysomnography or cardiorespiratory polygraphy, and the impact of different diagnostic methods has been previously discussed (30). In our study, differences between polysomnography and cardiorespiratory polygraphy were not taken into account either at baseline or at follow-up, as this would have caused a fragmentation of the sample in multiple subgroups complicating statistical analysis and decreasing statistical power. On the other hand, data at follow-up were obtained by sleep studies in many patients, whereas other studies relied exclusively on data downloaded from CPAP machines (12). Third, the database does not include depression among the recorded comorbidities. Depression is recognized as a major risk factor for EDS in CPAP-treated patients (17). The use of drugs for depression was reported by a minority of patients (N05-Psycholeptic drugs by 2.6% and N06-Psychoanaleptics by 6.3% of the patients), and we cannot exclude that untreated mild depression might have affected the prevalence of EDS. Finally, the ESS provides a subjective assessment of EDS, and its reliability and repeatability have been critically discussed (31, 32). Objective tests to assess sleepiness would be hardly applicable in large samples, but differences in ESS scores pre- and posttreatment were quite large and above the clinically meaningful differences reported by other studies, making it likely that they reflect true changes in EDS before–after treatment. In a recent study assessing objective sleepiness in effectively CPAP-treated patients with persistent EDS, 19 of 29 subjects (65%) were sleepy or severely sleepy at the Multiple Sleep Latency Test (33). Moreover, in the APPLES Study, MWT data were obtained in addition to ESS after 6 months of CPAP treatment, with good agreement between the prevalence of subjective and objective EDS. ESS score >10 was reported by 22% of the sample, and prevalence of sleep latency <17 min at MWT was 23% (13).

In conclusion, our data provide an estimate of the current prevalence of persistent EDS in CPAP-treated patients, around 40% in the first 3 months and between 10 and 20% after that. Occurrence of sleepiness at baseline, CPAP adherence, sleep duration at follow-up, and the timing of the follow-up visit predicted explained 40% of the variance in persistent EDS. Assessment of persistent EDS should be at least after 3 months of CPAP treatment, as EDS resolves over time in half of the patients reporting EDS at the start of CPAP treatment.

## Data Availability Statement

The data analyzed in this study was obtained from the European Sleep Apnea Database (ESADA), the following licenses/restrictions apply: the dataset analyzed in this study is available upon reasonable request from the ESADA office. Requests to access these datasets should be directed to Ludger Grote, ESADA office, Sahlgrenska Academy, University of Gothenburg, Gothenburg, Sweden, ludger.grote@lungall.gu.se.

## Ethics Statement

The studies involving human participants were reviewed and approved by the Ethics Committee of all ESADA Centers. The analysis presented is retrospective and was performed on the anonymized database. The patients/participants provided their written informed consent to participate in this study.

## Author Contributions

MB, JP, CB, OM, and FF: conceptualization. TS, HH, JV, OB, GT, IB, AP, JK, and SM: data collection. LG: funding acquisition. MB, FC, SB, and FF: data analysis and statistics. MB, FC, CB, OM, and FF: writing—original draft. JP, JV, TS, OB, GT, IB, WM, AP, JK, HH, SM, and LG: writing—review and editing. All authors contributed to the article and approved the submitted version.

## Collaborators in the ESADA Project as of March 2021 (Authors of the Paper Are Not Included in This List)

**P. Steiropoulos**, Sleep Unit, Department of Pneumonology, Democritus University of Thrace, Alexandroupolis, Greece; **E. Petiet**, Multidisciplinary Sleep Disorders Centre, Antwerp University Hospital, University of Antwerp, Antwerp, Belgium; **I. Fietze, T. Penzel, Naima Laharnar**, Schlafmedizinisches Zentrum, Charité – Universitätsmedizin Berlin, Berlin, Germany; **Ondrej Ludka**, Department of Cardiology, International Clinical Research Center, University Hospital Brno, St. Ann's University Hospital, Brno, Czechia; **S. Schiza**, Sleep Disorders Unit, Department of Respiratory Medicine, Medical School, University of Crete, Crete, Greece; **S. Ryan**, Pulmonary and SleepDisorders Unit, St. Vincent'sUniversityHospital, Dublin, Ireland; **R. L. Riha**, Department of Sleep Medicine, Royal Infirmary Edinburgh, Edinburgh, Scotland; **J. Hedner, D. Zou**, Pulmonary Department, Sleep Disorders Center, Sahlgrenska University Hospital, Göteborg, Sweden; Center of Sleep and Wake Disorders, Sahlgrenska Academy, Gothenburg University, Göteborg, Sweden; **M. S. Tasbakan**, Department of Chest Diseases, Ege University, Izmir, Turkey; **J. Buskova**, Department of Sleep Medicine, National Institute of Mental Health, Klecany, Czechia; **P. Joppa**, Department of Respiratory Medicine and Tuberculosis, Faculty of Medicine, P. J. Safarik University, L. Pasteur University Hospital, Kosice, Slovakia; **R. Staats** Department of Respiratory Medicine, Hospital de Santa Maria, Lisbon, Portugal; **Dries Testelmans**, Sleep Disorders Centre, University Hospital Gasthuisberg, Leuven, Belgium; **Haralampos Gouveris**, ENT Department at Mainz University Hospital, Mainz, Germany; **C. Lombardi, G. Parati**, Department of Cardiovascular, Neural and Metabolic Sciences, Istituto Auxologico Italiano, IRCCS, St. Luke Hospital, Milan, Italy; Department of Medicine and Surgery, University of Milano-Bicocca, Milan, Italy; **M. Petitjean, G. Roisman**, Unité de Médecine du Sommeil, Hopital Antoine-Beclere, Clamart, France; **M. Drummond, M. van Zeller**, Pulmonology Department Hospital São João, Medicine Faculty of Porto University, Porto, Portugal; **S. Herkenrath, W. Randerath**, Sleep Disorders Centre, Pulmonary Clinic, Solingen, Germany; **Z. Dogas, T. Galic**, Department of Neuroscience, Sleep Medicine Center, University of Split School of Medicine, Split, Croatia; **U. Anttalainen** Division of Medicine, Department of Pulmonary Diseases, Turku University Hospital and Sleep Research Centre, Turku, Finland; Department of Pulmonary Diseases and Clinical Allergology, University of Turku, Turku, Finland; **P. Sliwinski, R. Plywaczewski**, 2nd Department of Respiratory Medicine, Institute of Tuberculosis and Lung Diseases, Warsaw, Poland.

## Conflict of Interest

MB has no conflict of interest with regard to the submitted work. Outside the work he has the following COI: Honoraria or consultation fees: Bioprojet and Eisai. JV has no conflict of interest with regard to the submitted work. Outside the work he has the following COI: Honoraria or consultation fees: Bioprojet. LG has no conflict of interest with regard to the submitted work. Outside the work he has the following COI: Honoraria or consultation fees: Philips, Resmed, Itamar, Fisher & Paykel, and Astra Zeneca. Research collaboration with Resmed, Desitin, Weinmann, Itamar, and Breas. LG is a co-owner of patents related to pharmacological treatment of OSA. WM has no conflict of interest with regard to the submitted work. Outside the work he has the following COI: Honoraria or consultation fees: Jazz Pharmaceuticals. JP has no conflict of interest with regard to the submitted work. Outside the work he has the following COI: Grants/research support: Resmed, Philips, Fisher and Paykel, Agiradom, Astra Zeneca, Mutualia (research support), Vitalaire Air Liquide Foundation (research support), SEFAM; Honoraria or consultation fees: Resmed, Philips, Agiradom, Astra Zeneca, JAZZ, ITAMAR, SEFAM, Bioprojet. The remaining authors declare that the research was conducted in the absence of any commercial or financial relationships that could be construed as a potential conflict of interest.

## Publisher's Note

All claims expressed in this article are solely those of the authors and do not necessarily represent those of their affiliated organizations, or those of the publisher, the editors and the reviewers. Any product that may be evaluated in this article, or claim that may be made by its manufacturer, is not guaranteed or endorsed by the publisher.
