# Supplementary material for: Excessive Daytime Sleepiness in Obstructive Sleep Apnea Patients Treated With Continuous Positive Airway Pressure: Data From the European Sleep Apnea Database
Source: Front Neurol. 2021 Aug 9;12:690008. doi: 10.3389/fneur.2021.690008 (PMC8381644; doi:10.3389/fneur.2021.690008)
Supplement: Supplementary file 1 [file Data_Sheet_1.docx]

E-supplement of the paper:

**Excessive daytime sleepiness in obstructive sleep apnea (OSA) patients treated with continuous positive airway pressure (CPAP): data from the European Sleep Apnea Database (ESADA)**

Bonsignore MR^1,3^, Pepin JL^2^, Cibella F^3^, Barbera CDG^1^, Marrone O^3^, Verbraecken J^4^, Saaresranta T^5^, Basoglu OK^6^, Trakada G^7^, Bouloukaki I^8^, McNicholas WT^9^, Bailly S^2^, Pataka A^10^, Kvamme JA^11^, Hein H^12^, Mihaicuta S^13^, Grote L^14, 15^, Fanfulla F^16^, on behalf of the ESADA Study Group^§^

Table E1 reports the analysis of patients with and without monitoring data at follow-up (total n=4852).

Table E2 reports the characteristics of patients using automatic and fixed CPAP (n=2190).

**Table E1. Baseline characteristics of patients without and with sleep monitoring data at follow-up**

| **Variable** | **No sleep monitoringatfollow-up(n=2663)** | **Sleep monitoring atfollow-up(n=2190)** | **p** |
| --- | --- | --- | --- |
| Age, years | 54.5±11.7 | 55.1±12.0 | 0.053 |
| Females, n (%) | 709 (26%) | 592 (26.1%) | 0.95 |
| BMI (kg/m^2^) | 32.8±6.1 | 33.4±6.6 | **0.001** |
| AHI (events/h) | 42.7±23.8 | 39.2±22.9 | **<0.0001** |
| ODI 3% (events/h) | 35.2±25.7 | 38.3±25.0 | **<0.0001** |
| Lowest SpO_2_ (%) | 78.7±9.0 | 76.8±9.5 | **<0.0001** |
| MeanSpO_2_ (%) | 92.4±3.1 | 91.8±3.3 | **<0.0001** |
| Time spent at SpO_2_<90%, %(median,[IQR]) | 4.7 [0-6-16.5] | 4.2 [0.7-13.4] | 0.34 |
| ESS score | 10.4±5.2 | 10.2±5.1 | 0.06 |
| Subjectivesleepduration (h) | 6.6±1.7 | 6.9±1.4 | **<0.0001** |
| Subjectivesleeplatency (min) | 27.3±28.6 | 17.8±21.2 | **<0.0001** |
| CoronaryArterydisease (%) | 9.2% | 8.1% | 0.20 |
| SystemicHypertension (%) | 51.8% | 50.7% | 0.43 |
| Type 2 Diabetes(%) | 18.0% | 13.0% | **<0.0001** |
| COPD (%) | 6.5% | 7.3% | 0.27 |
| Insomnia (%) | 2.6% | 1.9% | 0.10 |
| Psychiatricdisease (%) | 10.2% | 4.5% | **<0.0001** |
| Drug treatment (%) | 77.0% | 46.2% | **<0.0001** |
| Follow-up duration (median, [IQR]) | 6 [4-12] | 3 [1-15] | 0.34 |

Comparison by unpaired t-test and X^2^ for continuous and categorical variables, respectively

Table E2. Comparison of patients according to prescription of automatic or fixed CPAP

| **Variable** | **Automatic CPAP(n=1776)** | **Fixed CPAP (n=414)** | **p** |
| --- | --- | --- | --- |
| Age, years | 54.9±11.8 | 56.3±12.6 | **0.023** |
| Females, n (%) | 443 (24.9%) | 128 (30.9%) | **0.01** |
| BMI (kg/m^2^) | 33.6±6.6 | 32.5±6.6 | **0.003** |
| AHI (events/h) | 39.3±23.1 | 38.6±22.0 | 0.58 |
| ODI 3% (events/h) | 38.8±24.8 | 36.0±25.5 | **0.04** |
| Lowest SaO_2_ (%) | 78.7±9.3 | 77.2±10.2 | 0.38 |
| MeanSaO_2_ (%) | 91.7±3.2 | 92.0±3.5 | 0.18 |
| Time Spent at SaO_2_<90%, % (median,[IQR]) | 4.6 [0.8-14.0] | 2.9 [0.4-10.2] | 0.34 |
| ESS Score | 10.2±5.1 | 10.2±5.2 | 0.91 |
| SubjectiveSleepDuration (h) | 6.8±1.4 | 7.2±1.3 | **<0.0001** |
| SubjectiveSleepLatency (min) | 17.6±21.1 | 19.3±21.9 | 0.25 |
| CoronaryArteryDisease (%) | 7.3% | 11.7% | **0.003** |
| SystemicHypertension (%) | 51.0% | 49.6% | 0.63 |
| Type 2 Diabetes(%) | 13.4% | 11.4% | 0.28 |
| COPD (%) | 7.5% | 6.6% | 0.51 |
| Insomnia (%) | 1.6% | 3.2% | **0.03** |
| PsychiatricDisease (%) | 4.1% | 6.3% | **0.045** |
| DrugTreatment (%) | 43.9% | 55.7% | **<0.0001** |
| Follow-up Duration (median, [IQR]) | 2 [0-15.5] | 6 [3-13] | 0.34 |
